# Comparative Effectiveness and Safety of Thulium Laser Enucleation and Holmium Laser Enucleation for BPH: Systematic Review and Meta-Analysis of Randomized Controlled Trials

**DOI:** 10.3390/jcm15176661

**Published:** 2026-08-28

**Authors:** Yuan Tang, Bo Xiong, Lin Yang, Changjian Zheng

**Affiliations:** 1 Department of Urology, The People’s Hospital of Tongnan District Chongqing City, Chongqing 402660, China; 2 Department of Urology, Bishan Hospital of Chongqing Medical University, Chongqing 402760, China

**Keywords:** holmium laser enucleation, thulium laser enucleation, thulium fiber laser, benign prostatic hyperplasia, systematic review

## Abstract

**Background**: Holmium laser enucleation of the prostate (HoLEP) is a minimally invasive surgery for BPH. Thulium-based techniques (ThuLEP/ThuFLEP) are increasingly used for their cutting/coagulation benefits, but their comparative outcomes versus HoLEP remain uncertain. **Methods**: Our synthesis relied solely on RCT data comparing ThuLEP/ThuFLEP versus HoLEP for BPH. Eligible studies were RCTs directly comparing ThuLEP or ThuFLEP with HoLEP. **Results**: Eight RCTs involving 1196 patients were included. Compared with HoLEP, thulium laser enucleation demonstrated similar perioperative outcomes, with no statistically significant differences observed in operative time, enucleated tissue weight, hospital stay, or catheterization time. Postoperative hemoglobin decrease was comparable between the two groups (MD −0.34, 95% CI −0.69 to 0.01, *p* = 0.05). Subgroup analyses indicated that ThuLEP might be associated with potential advantages in postoperative hemoglobin decrease and transient urinary incontinence; however, these findings require cautious interpretation due to limited sample sizes and heterogeneity among studies. No significant differences were identified in postoperative complications or functional recovery outcomes. **Conclusions**: Current evidence from RCTs indicates that thulium laser enucleation techniques and HoLEP provide similar efficacy and safety outcomes for patients undergoing surgical treatment of BPH.

## 1. Introduction

Lower urinary tract symptoms (LUTS) and bladder outlet obstruction are commonly seen in aging men with benign prostatic hyperplasia (BPH), leading to deterioration in patients’ quality of life [1,2]. For individuals who experience persistent symptoms or inadequate improvement after medical therapy, surgical treatment remains an important therapeutic option.

Historically, open prostatectomy was considered an effective surgical approach for BPH, particularly in patients with substantial prostate enlargement. Over time, the benefits of transurethral resection of the prostate (TURP) steadily established it as the gold-standard surgical intervention in routine practice, including reduced surgical trauma, decreased postoperative discomfort, and shorter hospitalization duration [3,4]. With continuous improvements in endoscopic and laser technologies, laser enucleation procedures have become increasingly important in the surgical management of BPH. Holmium laser enucleation of the prostate (HoLEP) has been widely adopted as an effective alternative to TURP owing to its reliable tissue removal capability, favorable hemostatic performance, and durable functional outcomes.

In recent years, thulium-based enucleation techniques include conventional thulium laser enucleation (ThuLEP) and thulium fiber laser enucleation (ThuFLEP). Although both utilize thulium-related laser technology, they differ in laser source characteristics and energy delivery patterns, which may influence surgical performance and postoperative outcomes [5,6].

Although both HoLEP and thulium laser enucleation techniques have been increasingly applied in clinical practice, their relative advantages remain uncertain. Previous systematic reviews and meta-analyses have attempted to compare these surgical approaches; however, some limitations remain. Several previous analyses incorporated non-randomized comparative studies, which may have been influenced by selection bias, differences in surgical expertise, and variations among institutions. Furthermore, previous evaluations limited to randomized controlled trials included relatively few studies, which restricted statistical power and prevented comprehensive subgroup analyses.

Since the publication of previous reviews, additional randomized controlled trials have been published, providing new evidence regarding the comparative effectiveness and safety of these procedures. In particular, newly available RCTs conducted between 2022 and 2024 have expanded the evidence base and enabled a more comprehensive assessment of potential differences between ThuLEP/ThuFLEP and HoLEP.

Therefore, this research aimed to compare surgical outcomes between thulium laser enucleation techniques (ThuLEP and ThuFLEP) and HoLEP based exclusively on randomized controlled trials.

## 2. Materials and Methods

### 2.1. Protocol and Registration

This meta-analysis was designed in accordance with PRISMA guidelines and the Cochrane Handbook recommendations [7,8]. Before data collection, we prospectively registered the study protocol with PROSPERO (CRD42024538001).

### 2.2. Search Strategy

We systematically searched PubMed, the Cochrane Library, Web of Science, Embase, Scopus, and the Chinese Biomedical Literature Database. Eligible publications available from database inception to August 2024 were considered. The search strategy was developed by combining terms describing the surgical techniques of interest (HoLEP, ThuLEP, and ThuFLEP), the target disease (BPH), and RCT designs. We also manually screened the reference lists of the included articles and related reports to identify additional eligible studies. The complete database-specific search strategies are provided in Supplementary Table S1.

The search strategy was structured around three core concepts, combined using the Boolean operator AND: (1) Laser interventions: MeSH terms and free-text keywords included “Laser Therapy” [MeSH], “Lasers, Solid-State” [MeSH], and procedural terms (holmium laser enucleation of the prostate, HoLEP; thulium laser enucleation of the prostate, ThuLEP; thulium fiber laser enucleation of the prostate, ThuFLEP). (2) Target condition: MeSH and text words comprised “Prostatic Hyperplasia” [MeSH], “Benign prostatic hyperplasia”, BPH, “prostatic hyperplasia”, and “lower urinary tract symptoms” (including LUTS in titles/abstracts, [tiab]). (3) Study design: We applied the filter “Randomized Controlled Trial”, along with randomized and RCT to restrict our search results to randomized evidence.

### 2.3. Eligibility Criteria

Enrolment criteria were established concerning study design, population, intervention, comparator, and outcomes. Eligible studies were RCTs enrolling adults with BPH and directly comparing ThuLEP/ThuFLEP with HoLEP, with at least one relevant clinical outcome reported. Non-original investigations were not considered for the final synthesis, including all forms of reviews, editorials, case reports, animal studies, conference abstracts without reported outcomes, non-RCTs, duplicate reports, studies involving prostate cancer patients, and studies unsuitable for quantitative synthesis.

### 2.4. Study Selection and Data Extraction

Two reviewers (Y. Tang and C. Zheng) independently screened the retrieved records against the pre-established inclusion criteria and abstracted the outcome data onto standardized extraction forms. Any disagreements were first discussed; if no agreement could be reached, a third reviewer was brought in to settle the matter. Extracted information included study characteristics, patient baseline data, laser technique, follow-up duration, surgical outcomes, and postoperative complications. Missing information was obtained by contacting authors when necessary. Continuous variables reported as medians and ranges were converted to means and standard deviations according to Luo et al. and Wan et al. [9,10].

### 2.5. Risk of Bias Assessment

Each included study underwent independent methodological quality appraisal by Y. Tang and C. Zheng employing the updated RoB 2 framework [11], which examined five core aspects (randomization, intervention deviations, missing outcome data, outcome measurement, and selective reporting); whenever the two reviewers disagreed, they reached a consensus through discussion.

### 2.6. Statistical Analysis

Meta-analyses were performed using Review Manager 5 (RevMan, version 5.4.1; The Cochrane Collaboration, Copenhagen, Denmark). The certainty of evidence was assessed using the GRADEpro Guideline Development Tool (GRADEpro GDT; McMaster University and Evidence Prime Inc., Hamilton, ON, Canada; https://www.gradepro.org). For continuous variables, pooled effects were expressed as mean differences (MDs) with 95% confidence intervals (CIs). Dichotomous outcomes were summarized as risk ratios (RRs) with corresponding 95% CIs. Statistical heterogeneity was assessed using Cochran’s Q test and the I^2^ statistic. A random-effects model was selected when considerable heterogeneity existed (I^2^ > 50%), whereas a fixed-effects model was applied for outcomes without substantial heterogeneity. The stability of pooled results was examined through sequential removal of individual studies. We assessed potential publication bias via funnel plots and Egger’s regression test, provided that an adequate number of studies were obtainable.

### 2.7. GRADE Evidence Assessment

For each outcome, the certainty rating (high, moderate, low, or very low) was derived from GRADE evaluations of five potential sources of bias—including risk of bias, inconsistency, indirectness, imprecision, and publication bias—and was accompanied by a table summarizing the graded evidence.

## 3. Results

### 3.1. Characteristics of the Included Trials and Their Selection Pathway

**Figure 1:**
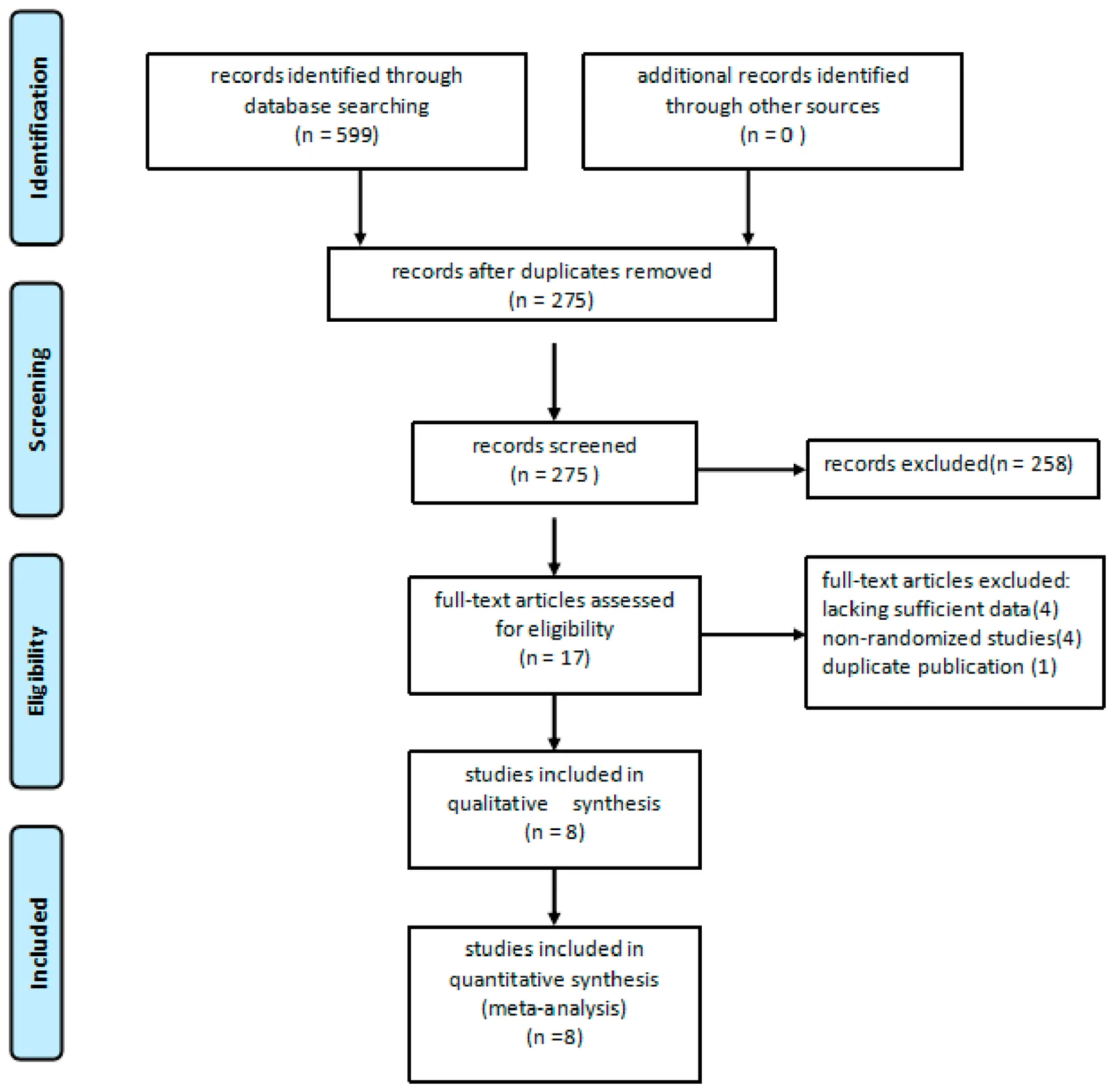
PRISMA flow diagram.

Only 17 articles ultimately qualified for comprehensive full-text assessment, a number distilled from 599 raw database hits after excluding duplicates and ruling out irrelevant entries through an initial triage. Nine studies were subsequently excluded because of unavailable extractable data (n = 4), non-randomized designs (n = 4), or duplicate reporting (n = 1). Finally, eight RCTs published from 2012 to 2024 were included in the quantitative synthesis [12,13,14,15,16,17,18,19]. These trials enrolled 1196 patients with BPH and directly compared thulium laser enucleation (ThuLEP or ThuFLEP) with HoLEP. Table 1 gives a concise summary of the principal study attributes. The study-selection process is shown in Figure 1. The complete database-specific search strategies are provided in Table S2.

### 3.2. Risk of Bias and Certainty of Evidence

**Figure 2:**
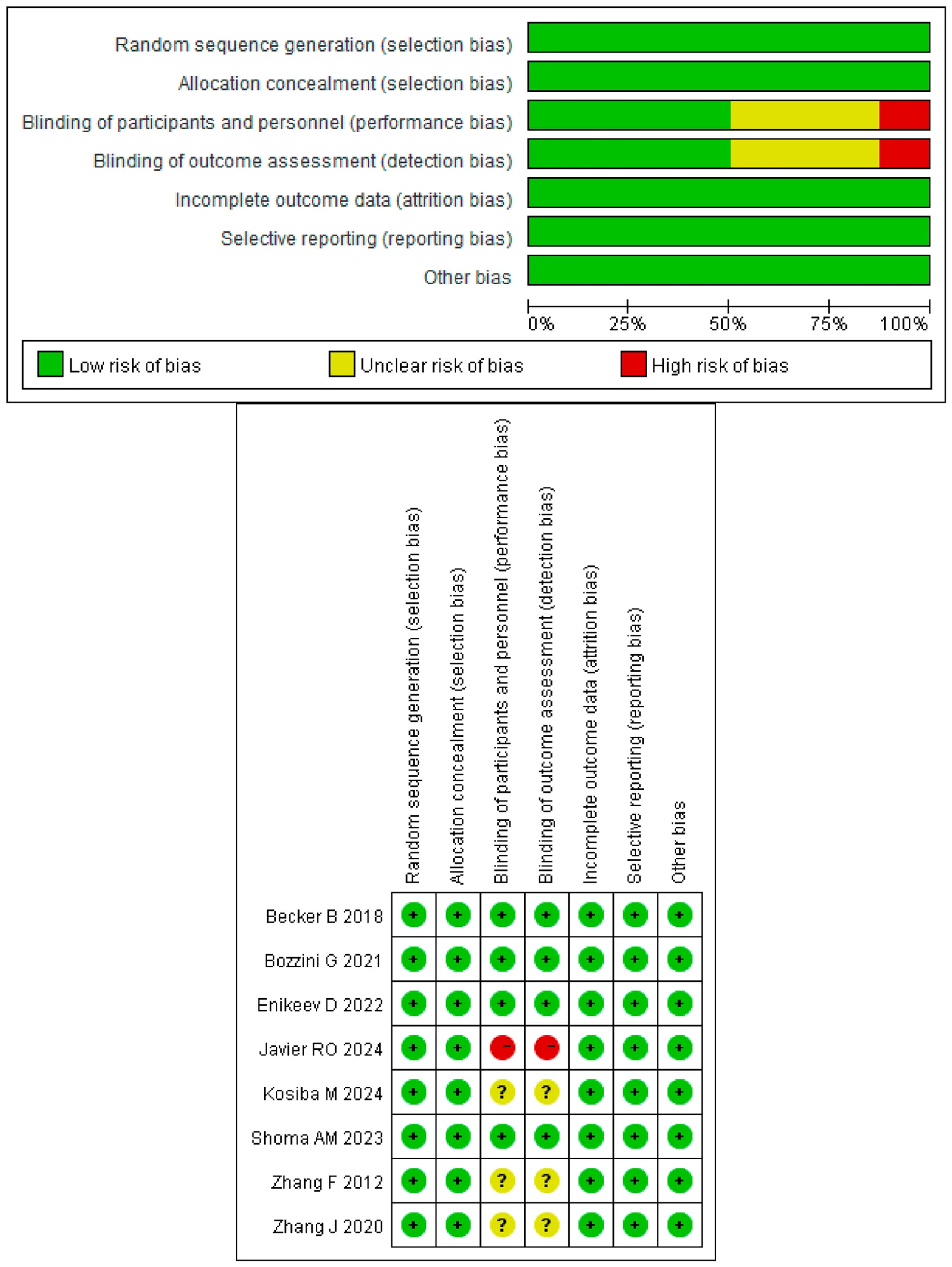
Risk-of-bias assessment of the included randomized controlled trials [12,13,14,15,16,17,18,19].

Table 2 details the GRADE-based evidence certainty (moderate to high) for the evaluated outcomes. As for methodological quality, the risk-of-bias assessment deemed the included trials acceptable, with low-to-moderate bias risk overall. All trials furnished reports on random sequence generation. The risk-of-bias assessments are presented in Figure 2.

### 3.3. Meta-Analysis

#### 3.3.1. Operative Time

**Figure 3:**
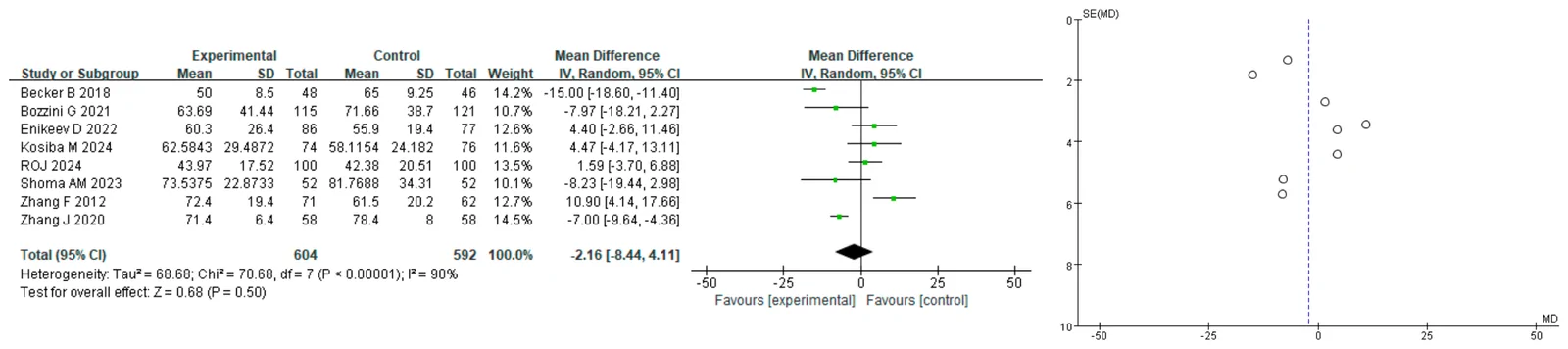
Forest plot and funnel plot of operative time in the meta-analysis [12,13,14,15,16,17,18,19]. Squares and horizontal lines represent study-specific MDs and 95% CIs, respectively; diamonds indicate pooled estimates, and the solid vertical line indicates no effect (MD = 0). In funnel plots, circles represent individual studies, while dashed lines indicate pooled estimates and, where shown, approximate 95% confidence limits.

A random-effects model was employed for the eight studies (n = 1196) reporting operative time [12,13,14,15,16,17,18,19], given the considerable heterogeneity (I^2^ = 90%). The substantial heterogeneity may reflect differences in surgical expertise, laser parameters, and procedural techniques among the included RCTs. The pooled analysis demonstrated no statistically significant difference in operative time between thulium laser enucleation and HoLEP: MD −2.16, 95% CI −8.44 to 4.11, *p* = 0.50 (Figure 3). Subgroup analyses showed no significant differences between ThuLEP and HoLEP (MD −5.54, 95% CI −13.49 to 2.42, *p* = 0.17) or between ThuFLEP and HoLEP (MD 2.96, 95% CI −0.84 to 6.67, *p* = 0.13). Sensitivity analyses confirmed the robustness of the pooled estimates, with no evidence of publication bias (*p* > 0.05) and moderate evidence certainty (Table 2).

#### 3.3.2. Enucleation Weight

**Figure 4:**
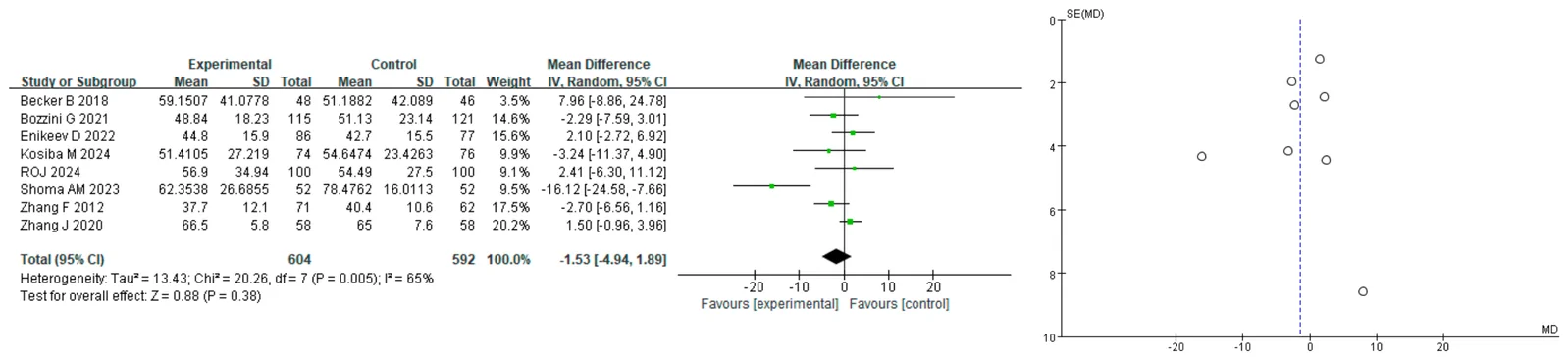
Forest plot and funnel plot of enucleated weight in the meta-analysis [12,13,14,15,16,17,18,19]. Squares and horizontal lines represent study-specific MDs and 95% CIs, respectively; diamonds indicate pooled estimates, and the solid vertical line indicates no effect (MD = 0). In funnel plots, circles represent individual studies, while dashed lines indicate pooled estimates and, where shown, approximate 95% confidence limits.

Eight studies reported postoperative enucleation tissue weight [12,13,14,15,16,17,18,19]. We found no statistically significant difference between thulium laser enucleation and HoLEP: MD −1.53, 95% CI −4.94 to 1.89, *p* = 0.38 (Figure 4). Subgroup analyses showed no significant difference between ThuLEP and HoLEP (MD −2.88, 95% CI −7.93 to 2.17, *p* = 0.26) or ThuFLEP and HoLEP (MD 1.03, 95% CI −2.72 to 4.77, *p* = 0.59). Sensitivity analyses confirmed the robustness of the pooled estimates, with no evidence of publication bias (*p* > 0.05) and moderate evidence certainty (Table 2).

#### 3.3.3. Hospital Stay

**Figure 5:**
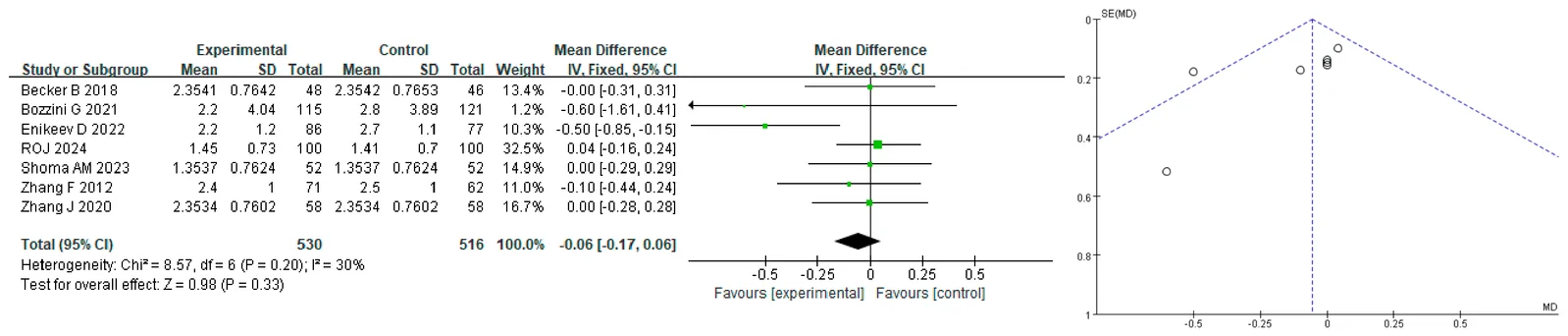
Forest plot and funnel plot of hospital stay in the meta-analysis [12,14,15,16,17,18,19]. Squares and horizontal lines represent study-specific MDs and 95% CIs, respectively; diamonds indicate pooled estimates, and the solid vertical line indicates no effect (MD = 0). In funnel plots, circles represent individual studies, while dashed lines indicate pooled estimates and, where shown, approximate 95% confidence limits.

Seven studies reported postoperative hospital stay outcomes [12,14,15,16,17,18,19]. No significant heterogeneity was observed, and combined estimates showed no significant difference: MD −0.06, 95% CI −0.17 to 0.06, *p* = 0.33 (Figure 5). Subgroup analyses also demonstrated no significant differences between ThuLEP and HoLEP (MD −0.03, 95% CI −0.18 to 0.12, *p* = 0.67) or between ThuFLEP and HoLEP (MD −0.21, 95% CI −0.74 to 0.009, *p* = 0.44). Potential publication bias (*p* < 0.05) prompted us to downgrade evidence certainty to moderate (GRADE, Table 2), while sensitivity analyses yielded stable pooled estimates.

#### 3.3.4. Catheterization Time

**Figure 6:**
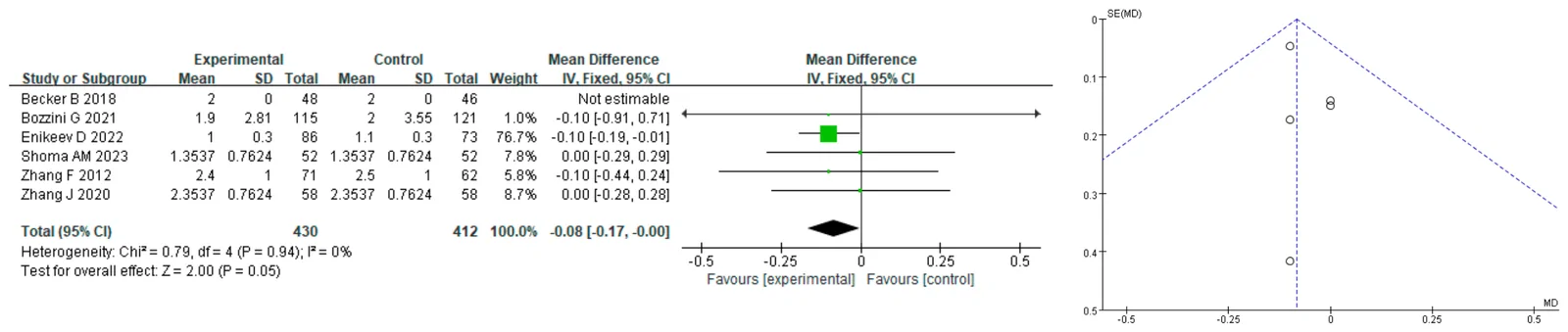
Forest plot and funnel plot of catheterization time in the meta-analysis [14,15,16,17,18,19]. Squares and horizontal lines represent study-specific MDs and 95% CIs, respectively; diamonds indicate pooled estimates, and the solid vertical line indicates no effect (MD = 0). In funnel plots, circles represent individual studies, while dashed lines indicate pooled estimates and, where shown, approximate 95% confidence limits.

Six studies evaluated catheterization time [14,15,16,17,18,19]. The lack of significant statistical heterogeneity justified the use of a fixed-effects model. We found no statistically significant difference between groups: MD −0.08, 95% CI −0.17 to 0.00, *p* = 0.05 (Figure 6). Although the result approached statistical significance, the confidence interval included zero, indicating that the observed difference should be interpreted as a possible trend rather than confirmed superiority. Subgroup analysis showed no significant difference between ThuLEP and HoLEP. For ThuFLEP, catheterization time was shorter compared with HoLEP: MD −0.10, 95% CI −0.19 to −0.01, *p* = 0.04. However, this finding requires cautious interpretation because of the limited number of available studies. The evidence received a high certainty rating (Table 2).

#### 3.3.5. Hemoglobin Decrease

**Figure 7:**
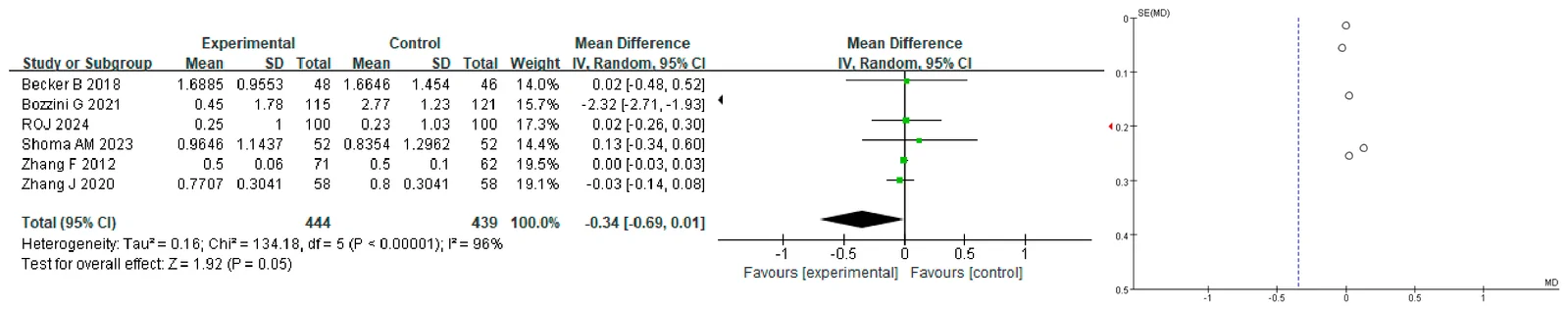
Forest plot and funnel plot of hemoglobin decrease in the meta-analysis [12,14,16,17,18,19]. Squares and horizontal lines represent study-specific MDs and 95% CIs, respectively; diamonds indicate pooled estimates, and the solid vertical line indicates no effect (MD = 0). In funnel plots, circles represent individual studies, while dashed lines indicate pooled estimates and, where shown, approximate 95% confidence limits.

Six studies reported postoperative hemoglobin decrease outcomes. We found no statistically significant difference between thulium laser enucleation and HoLEP: MD −0.34, 95% CI −0.69 to 0.01, *p* = 0.05 (Figure 7). Although the pooled estimate indicated a possible tendency toward reduced hemoglobin decrease with thulium laser enucleation, the confidence interval crossed zero, suggesting that the difference did not reach definitive statistical significance. Subgroup analysis demonstrated that ThuLEP was associated with a smaller postoperative hemoglobin decrease compared with HoLEP: MD −0.42, 95% CI −0.83 to −0.01, *p* = 0.04. However, no significant difference was observed between ThuFLEP and HoLEP: MD −0.02, 95% CI −0.26 to 0.30, *p* = 0.89. Heterogeneity and potential publication bias (*p* < 0.05) prompted us to downgrade evidence certainty to moderate (GRADE, Table 2), while sensitivity analyses yielded stable pooled estimates (Table 2).

#### 3.3.6. Transient Urinary Incontinence

**Figure 8:**
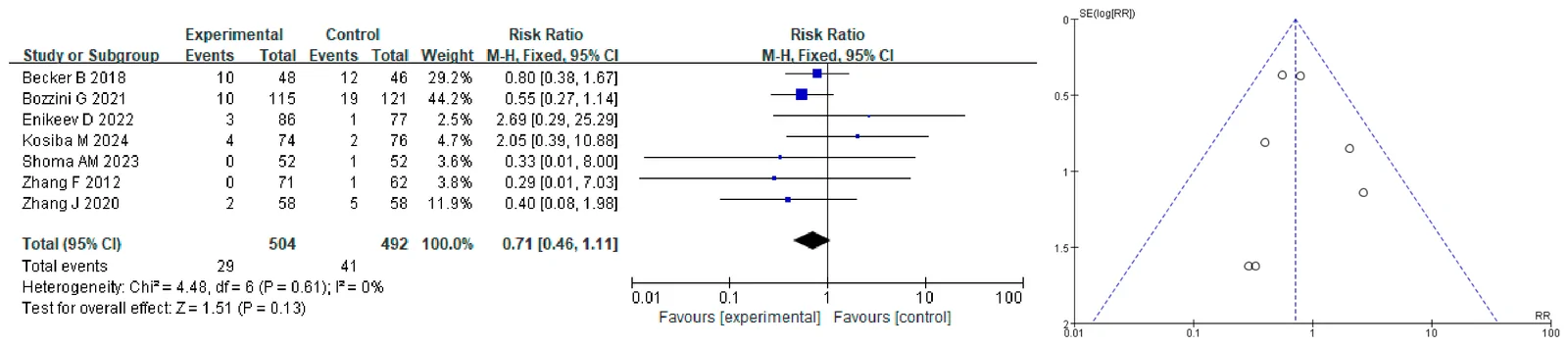
Forest plot and funnel plot of transient urinary incontinence in the meta-analysis [13,14,15,16,17,18,19]. Squares and horizontal lines represent study-specific RRs and 95% CIs, respectively; diamonds indicate pooled RRs, and the solid vertical line indicates no effect (RR = 1). In funnel plots, circles represent individual studies, while dashed lines indicate pooled estimates and, where shown, approximate 95% confidence limits.

Seven studies reported postoperative transient urinary incontinence outcomes [13,14,15,16,17,18,19]. We found no statistically significant difference between thulium laser enucleation and HoLEP: RR = 0.71, 95% CI 0.46 to 1.11, *p* = 0.13 (Figure 8). No significant heterogeneity was detected among the included studies (I^2^ = 0%); therefore, a fixed-effects model was applied. Subgroup analysis suggested that ThuLEP was associated with a lower incidence of transient urinary incontinence compared with HoLEP: RR = 0.59, 95% CI 0.37 to 0.95, *p* = 0.03. In contrast, no statistically significant difference was observed between ThuFLEP and HoLEP: RR = 2.27, 95% CI 0.60 to 8.66, *p* = 0.23. Sensitivity analyses confirmed the robustness of the pooled estimates, with no evidence of publication bias (*p* > 0.05) and high evidence certainty (Table 2).

#### 3.3.7. Complications

**Figure 9:**
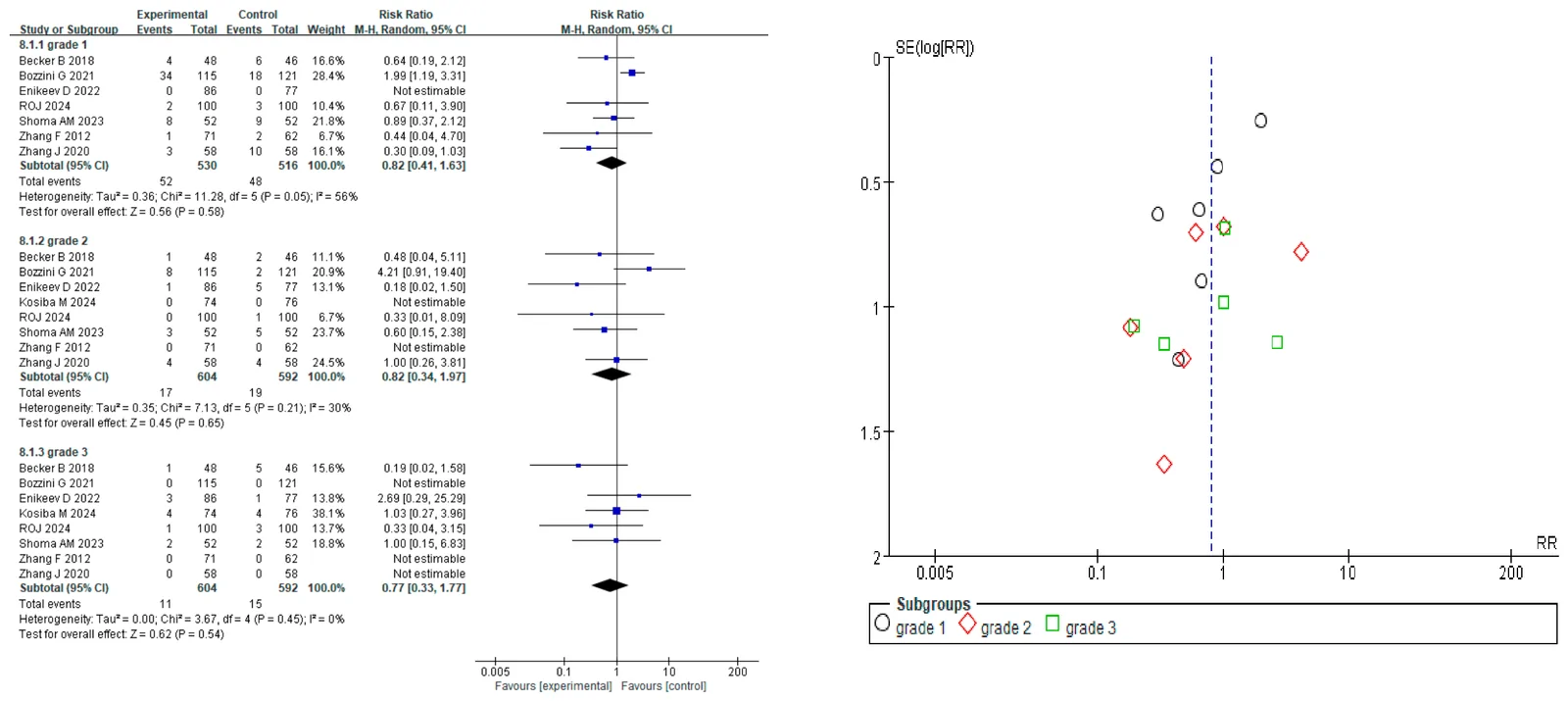
Forest plot and funnel plot of complications in the meta-analysis [12,13,14,15,16,17,18,19]. Squares and horizontal lines represent study-specific RRs and 95% CIs, respectively; diamonds indicate pooled RRs, and the solid vertical line indicates no effect (RR = 1). In funnel plots, circles represent individual studies, while dashed lines indicate pooled estimates and, where shown, approximate 95% confidence limits.

Eight studies reported postoperative complications [12,13,14,15,16,17,18,19]. Combined estimates showed no significant differences between thulium laser enucleation and HoLEP for different grades of complications: Clavien–Dindo grade I complications: RR = 0.82, 95% CI 0.41 to 1.63, *p* = 0.58; Clavien–Dindo grade II complications: RR = 0.82, 95% CI 0.34 to 1.97, *p* = 0.65; Clavien–Dindo grade III complications: RR = 0.77, 95% CI 0.33 to 1.77, *p* = 0.54 (Figure 9). Subgroup analyses demonstrated comparable complication rates between ThuLEP and HoLEP (Clavien–Dindo grade I complications: RR = 0.82, 95% CI 0.38 to 1.78, *p* = 0.62; Clavien–Dindo grade II complications: RR = 1.13, 95% CI 0.44 to 2.88, *p* = 0.79; Clavien–Dindo grade III complications: RR = 0.46, 95% CI 0.09 to 2.37, *p* = 0.36) or ThuFLEP and HoLEP (Clavien–Dindo grade I complications: RR = 0.67, 95% CI 0.11 to 3.9, *p* = 0.65; Clavien–Dindo grade II complications: RR = 0.22, 95% CI 0.04 to 1.27, *p* = 0.09; Clavien–Dindo grade III complications: RR = 0.99, 95% CI 0.36 to 2.77, *p* = 0.99). Sensitivity analyses confirmed the robustness of the pooled estimates, with no evidence of publication bias (*p* > 0.05) and moderate evidence certainty (Table 2).

#### 3.3.8. Qmax

**Figure 10:**
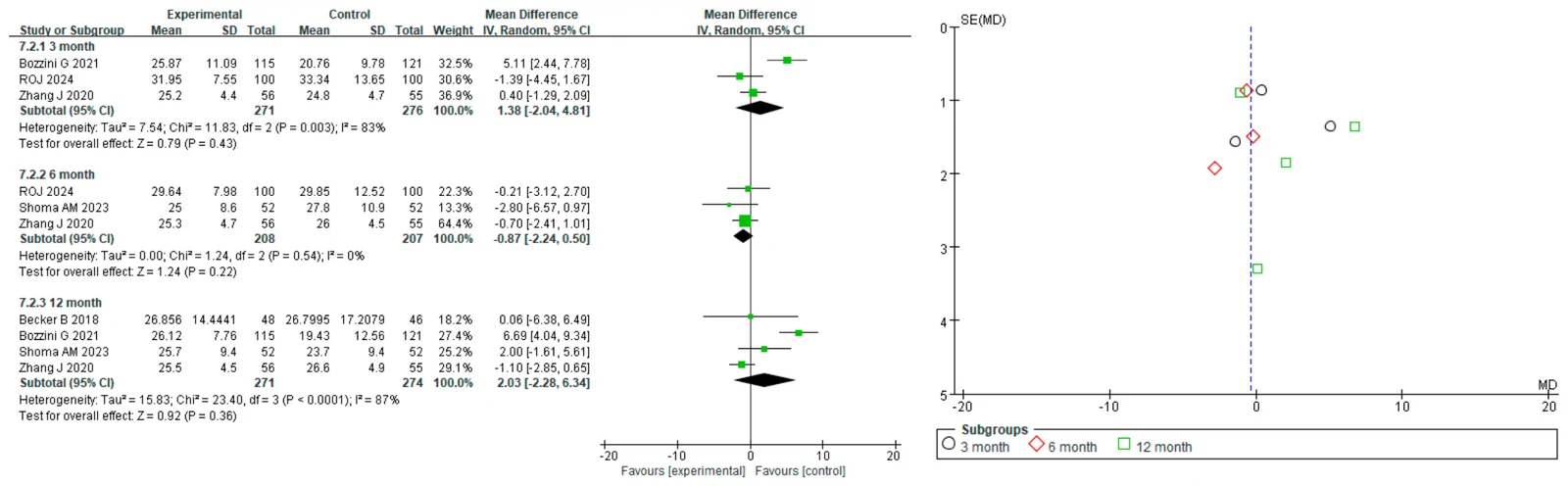
Forest plot and funnel plot comparing postoperative maximum urinary flow rate (Qmax) in the meta-analysis [14,16,17,18,19]. Squares and horizontal lines represent study-specific MDs and 95% CIs, respectively; diamonds indicate pooled estimates, and the solid vertical line indicates no effect (MD = 0). In funnel plots, circles represent individual studies, while dashed lines indicate pooled estimates and, where shown, approximate 95% confidence limits.

Postoperative Qmax was evaluated in four studies [14,16,17,18,19]. The follow-up periods ranged from 3 to 18 months. No statistically significant differences were observed between thulium laser enucleation and HoLEP at different follow-up time points: 3 months: MD 1.38, 95% CI −2.04 to 4.81, *p* = 0.43; 6 months: MD −0.87, 95% CI −2.24 to 0.50, *p* = 0.22; 12 months: MD 2.03, 95% CI −2.28 to 6.34, *p* = 0.36 (Figure 10). Subgroup analysis showed no significant differences between ThuLEP and HoLEP (MD 0.05, 95% CI −1.24 to 1.34, *p* = 0.94) or ThuFLEP and HoLEP (MD 0.45, 95% CI −1.82 to 2.72, *p* = 0.70). Sensitivity analysis showed consistent results. No significant publication bias was detected (*p* > 0.05). The certainty of evidence was rated as moderate (Table 2).

#### 3.3.9. Enucleation Time

**Figure 11:**
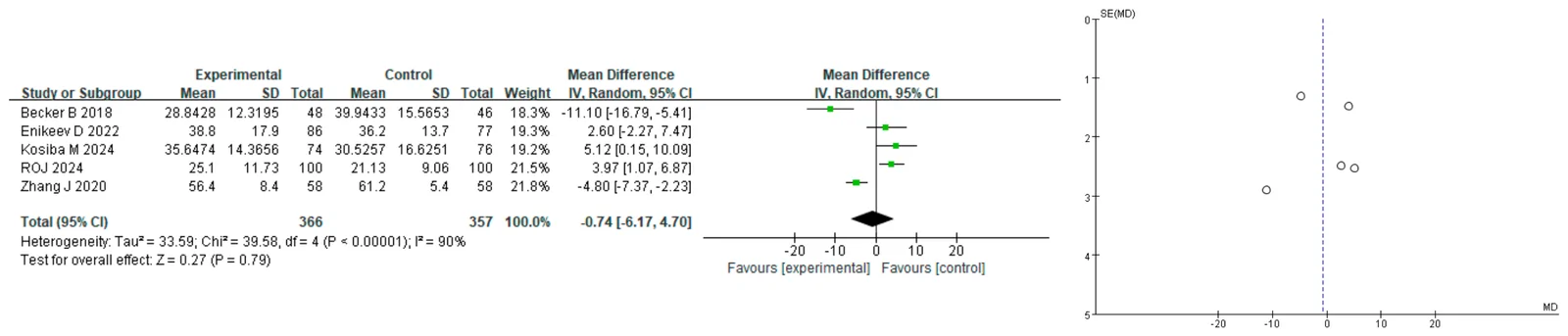
Forest plot and funnel plot of enucleation time in the meta-analysis [12,13,15,17,18]. Squares and horizontal lines represent study-specific MDs and 95% CIs, respectively; diamonds indicate pooled estimates, and the solid vertical line indicates no effect (MD = 0). In funnel plots, circles represent individual studies, while dashed lines indicate pooled estimates and, where shown, approximate 95% confidence limits.

Five studies reported enucleation time outcomes [12,13,15,17,18]. Given the considerable between-study variability, we adopted a random-effects approach. The pooled analysis showed no significant difference between thulium laser enucleation and HoLEP: MD −0.74, 95% CI −6.17 to 4.7, *p* = 0.79 (Figure 11). Subgroup analysis showed no significant differences between ThuLEP and HoLEP or between ThuFLEP and HoLEP. The observed heterogeneity may be related to differences in laser platforms, surgeon experience, operative techniques, or patient characteristics. Sensitivity analyses confirmed the robustness of the pooled estimates, with no evidence of publication bias (*p* > 0.05) and moderate evidence certainty (Table 2).

#### 3.3.10. Morcellation Time

**Figure 12:**
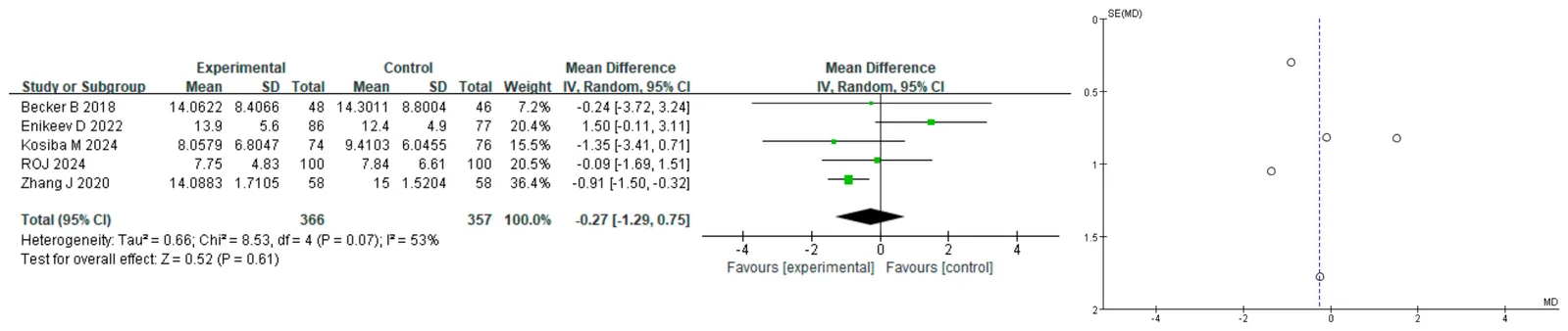
Forest plot and funnel plot of morcellation time in the meta-analysis [12,13,15,17,18]. Squares and horizontal lines represent study-specific MDs and 95% CIs, respectively; diamonds indicate pooled estimates, and the solid vertical line indicates no effect (MD = 0). In funnel plots, circles represent individual studies, while dashed lines indicate pooled estimates and, where shown, approximate 95% confidence limits.

Five studies reported morcellation time outcomes [12,13,15,17,18]. Combined estimates showed no significant difference between thulium laser enucleation and HoLEP: MD −0.27, 95% CI −1.29 to 0.75, *p* = 0.61 (Figure 12). Subgroup analysis showed significant differences between ThuLEP and HoLEP (MD −0.89, 95% CI −1.47 to −0.31, *p* = 0.003) but no significant differences between ThuFLEP and HoLEP (MD 0.11, 95% CI −1.45 to 1.68, *p* = 0.89). The overall estimate was not materially altered by omitting any one study, as per our sensitivity analysis. No evidence of significant publication bias was identified (*p* > 0.05). The certainty of evidence was rated as moderate (Table 2).

## 4. Discussion

In our meta-analysis, we included eight RCTs involving 1196 patients to compare thulium laser enucleation techniques with HoLEP for the treatment of BPH. The pooled results demonstrated that thulium laser enucleation and HoLEP provided comparable perioperative outcomes, including operative time, enucleated tissue weight, hospital stay, catheterization time, postoperative hemoglobin decrease, postoperative complications, and functional outcomes. Although subgroup analyses suggested potential differences between ThuLEP and ThuFLEP in some outcomes, the small sample sizes and clinical heterogeneity necessitate prudence in the interpretation of these observations. Importantly, statistical differences observed in individual outcomes should not automatically be interpreted as clinically meaningful advantages, particularly when the absolute effect sizes are small. Therefore, the overall clinical implication of our findings is that thulium-based enucleation techniques provide comparable efficacy and safety profiles rather than clear superiority over HoLEP.

The present study has several important differences compared with previously published systematic reviews and meta-analyses. Previous analyses have provided valuable evidence regarding the comparison between thulium laser enucleation and HoLEP; however, some included non-randomized studies, which may introduce potential bias related to patient selection, surgeon experience, and institutional differences. In contrast, our analysis focused exclusively on randomized controlled trials and incorporated several recently published RCTs, thereby increasing the available sample size and allowing further subgroup analyses according to different thulium laser technologies. These additional data provide a more comprehensive evaluation of the comparative efficacy and safety between ThuLEP/ThuFLEP and HoLEP.

Postoperative complications are important indicators for evaluating surgical safety. In the present study, complications were analyzed according to the available definitions reported in the included studies. The Clavien–Dindo classification provides a widely accepted framework for grading surgical complications and improves consistency in postoperative safety evaluation [20]. However, differences in complication definitions among individual studies may contribute to heterogeneity and should be considered when interpreting pooled results.

HoLEP, initially introduced by Gilling et al., has become an established endoscopic treatment option because of its durable efficacy and favorable safety profile [21]. With technological advances, thulium laser systems, including ThuLEP and ThuFLEP, have been increasingly adopted. Differences in laser wavelength, tissue interaction, and energy delivery mechanisms may theoretically influence cutting efficiency and coagulation performance [22]. Although both ThuLEP and ThuFLEP belong to thulium-based laser techniques, they are not completely interchangeable. Conventional thulium laser systems and thulium fiber lasers differ in laser source characteristics and energy delivery patterns, which may influence tissue effects and surgical performance [23]. However, current randomized evidence remains insufficient to determine whether one thulium platform provides clinically superior outcomes.

Previous studies have also investigated thulium fiber laser technology in urinary stone treatment. Although favorable outcomes have been reported in this field, the clinical application of laser lithotripsy differs substantially from prostate enucleation because of differences in target tissue characteristics and surgical objectives [24]. Therefore, evidence from stone management should not be directly extrapolated to BPH surgery.

Anatomical endoscopic enucleation has become an important surgical approach for BPH. Previous meta-analyses have demonstrated that enucleation techniques provide effective symptom relief and acceptable safety compared with conventional transurethral resection procedures [25]. The current findings further support that both thulium laser enucleation and HoLEP can achieve satisfactory short- to intermediate-term outcomes in appropriately selected patients.

Compared with previous meta-analyses specifically evaluating ThuLEP and HoLEP, Meng et al. reported potential perioperative advantages of thulium laser enucleation; however, differences in study design and inclusion criteria may have influenced their conclusions [26]. Subsequently, Hartung et al. conducted a meta-analysis focusing on RCTs and reported comparable efficacy and safety between the two techniques [27]. Our study extends previous evidence by incorporating additional RCTs published in recent years and performing further subgroup analyses according to laser type. Nevertheless, the available evidence remains insufficient to confirm the superiority of either technique.

Several factors may explain the heterogeneity observed in some outcomes. First, different thulium laser technologies were included, including conventional thulium laser systems and thulium fiber laser systems, which differ in energy delivery characteristics. Second, variations in laser settings, surgical techniques, surgeon experience, prostate size, and perioperative management protocols may influence outcomes. Although subgroup analyses were performed according to laser type, the limited number of available studies restricted the statistical power of these analyses. In addition, surgeon experience and learning curve effects may substantially influence outcomes of anatomical endoscopic enucleation. Previous studies have demonstrated that surgical efficiency improves with increasing procedural experience during laser enucleation procedures [28,29]. Since the included RCTs involved surgeons at different stages of experience, variations in operator proficiency may partially explain the observed heterogeneity.

Although some subgroup findings suggested potential advantages of ThuLEP regarding postoperative hemoglobin decrease and transient urinary incontinence, these results should be interpreted cautiously because of limited sample sizes and clinical heterogeneity. The observed statistical significance does not necessarily indicate a clinically meaningful benefit, considering the relatively small number of included trials and the potential influence of surgical technique, surgeon experience, and perioperative management. Therefore, current evidence supports comparable efficacy and safety between thulium-based enucleation and HoLEP rather than superiority of one technique.

The potential hemostatic advantage of thulium laser systems has been frequently discussed because of their physical characteristics. However, in our meta-analysis, postoperative hemoglobin decrease was not significantly different between thulium laser enucleation and HoLEP. Regarding postoperative hemoglobin decrease, although the pooled estimate favored ThuLEP, the absolute difference was modest. Whether this numerical reduction results in clinically relevant benefits, such as reduced transfusion risk or shorter hospitalization, remains uncertain. Therefore, this finding should be considered hypothesis-generating rather than evidence of superiority. For transient urinary incontinence, potential explanations may include differences in tissue interaction and surgical handling; however, continence outcomes are also strongly influenced by apical dissection technique, preservation of the external sphincter, and surgeon experience.

Long-term durability is another important consideration when evaluating surgical techniques for BPH. Previous observational studies have reported favorable long-term functional outcomes after ThuLEP, including sustained symptom improvement during extended follow-up periods [30]. Similarly, long-term follow-up studies have demonstrated acceptable durability and low retreatment rates after ThuLEP [31]. However, these findings were mainly derived from observational cohorts rather than randomized comparisons. Because most RCTs included in the present meta-analysis had short- to intermediate-term follow-up durations, the long-term comparative effectiveness between thulium laser enucleation and HoLEP remains uncertain.

This meta-analysis has several limitations. First, operator experience may have influenced the outcomes because all included studies involved surgical interventions. Among the included RCTs, six studies provided information regarding surgeon expertise, reporting a substantial difference between low-volume and high-volume surgeons (approximately 40 and 500 prior procedures, respectively). However, one study [18] did not describe the experience level of the operating surgeons, making it difficult to assess the potential impact of operator proficiency in that trial. Second, the available evidence regarding long-term erectile function was limited. Only one included study [13] reported long-term erectile function outcomes, highlighting the need for future research to further investigate this clinically important endpoint. Third, several sources may contribute to the observed heterogeneity, including differences in surgeon experience, learning curve stage, prostate volume, laser platforms, and perioperative management protocols. However, detailed information regarding these factors was inconsistently reported among included RCTs, preventing further quantitative analysis. Future multicenter RCTs with standardized surgical protocols are required to clarify these potential effects. In addition, the relatively small number of eligible studies and limited sample sizes restricted the strength of the current conclusions. Therefore, further well-designed, large-scale RCTs with standardized methodology and extended follow-up are required to provide more robust evidence and update the present findings.

## 5. Conclusions

In conclusion, current evidence indicates that both thulium laser enucleation and HoLEP are safe and effective surgical techniques for the treatment of BPH, with comparable perioperative and functional outcomes. Further large-scale, multicenter RCTs with standardized surgical protocols and longer follow-up periods are required to clarify the long-term efficacy, safety, and optimal clinical application of these laser enucleation techniques.

## Figures and Tables

**Table 1:** Basic characteristics of included studies.

Study (Years)	Country	Study		ThuLEP/ThuFLEP Group/HoLEP Group						
		Design	Group	Age (Year)	Patients	Qmax Baseline (mL/S)	IPSS Baseline	PVR (mL)	QoL Data	PSA Baseline
										(ng/mL)
Romero Otero J 2024 [12]	Spain	RCT	ThuFLEP	68.7 ± 7.90	100	10.46 ± 6.31	22.07 ± 7.68	146 ± 155.43	4.53 ± 1.37	5.22 ± 5.8
			HoLEP	69.61 ± 8.38	100	10.51 ± 4.71	23.14 ± 6.8	73 ± 75.08	4.85 ±0.89	4.24 ± 3.57
Kosiba M 2024 [13]	Germany	RCT	ThuFLEP	65	74	6.8–13.4	17–26	50–150	3.75–5	2.8–7.1
			HoLEP	74	76	6.7–11.9	16–25	40–200	3.5–5	2.9–8.7
Shoma AM 2023 [14]	Egypt	RCT	ThuLEP	62.25–72	52	11 ± 3.2	14–27	2.5–67.5	5.0–5	NA
			HoLEP	62–73	52	10.2 ± 2.6	21–27.5	13.8–104	4.0–5	
Enikeev D 2022 [15]	Russia	RCT	ThuFLEP	64.3 ± 6.2	86	8.7 ± 3.2	23.3 ± 6.0	76.5 ± 70.2	5.1 ± 0.8	3.8 ± 3.8
			HoLEP	65.0 ± 7.0	77	8.6 ± 2.6	24.3 ± 5.1	75.5 ± 77.3	5.0 ± 1.0	4.2 ± 4.1
Bozzini G 2021 [16]	Italy	RCT	ThuLEP	67.1 ± 17.83	115	7.9 ± 8.05	18.2 ± 7.31	115.5 ± 130.54	NA	3.2 ± 4.14
			HoLEP	69.5 ± 15.54	121	8.2 ± 6.71	17.9 ± 6.95	90.4 ± 120.44		2.9 ± 5.25
Zhang J 2020 [17]	China	RCT	ThuLEP	72.7 ± 3.1	58	6.6 ± 2.3	22.8 ± 3.7	165.5 ± 46.2	4.0–6	4.96 ± 1.40
			HoLEP	71.8 ± 3.9	58	7.1 ± 2.8	23.9 ± 3.9	172.7 ± 39.4	4.0–6	5.09 ± 1.49
Becker B 2018 [18]	Germany	RCT	ThuLEP	68–76.75	48	6.2–12.4	16–25	26.75–237.5	4.0–5	1.98–6.28
			HoLEP	67–75	46	7.2–15	15–26	48.25–200	4.0–5	2.18–8.37
Zhang F 2012 [19]	China	RCT	ThuLEP	76.2 ± 9.7	71	6.8 ± 3.9	24.6 ± 3.2	64. ± 32.5	NA	2.56 ± 2.19
			HoLEP	73.4 ± 10.3	62	7.3 ± 3.7	22.8 ± 2.6	64.6 ± 33.4		2.07 ± 2.22

Qmax: Maximum urinary flow rate; IPSS: International Prostate Symptom Score; PVR: postvoid residual urine volume; QoL: quality of life; NA: not available.

**Table 2:** GRADE certainty assessment.

Outcome	No. of Studies	Risk of Bias	Inconsistency	Indirectness	Imprecision	Publication Bias	Overall Certainty
Operative time	8	Not serious	Serious	Not serious	Not serious	Not suspected	⨁⨁⨁◯ Moderate
Enucleated tissue weight	8	Not serious	Serious	Not serious	Not serious	Not suspected	⨁⨁⨁◯ Moderate
Hospital stay	7	Not serious	Not serious	Not serious	Not serious	Not suspected	⨁⨁⨁⨁ High
Catheterization time	6	Not serious	Not serious	Not serious	Not serious	Not suspected	⨁⨁⨁⨁ High
Hemoglobin decrease	6	Not serious	Serious	Not serious	Not serious	Not suspected	⨁⨁⨁◯ Moderate
Transient urinary incontinence	7	Not serious	Not serious	Not serious	Serious	Not suspected	⨁⨁⨁◯ Moderate
Complications	8	Not serious	Not serious	Not serious	Serious	Not suspected	⨁⨁⨁◯ Moderate
Qmax	5	Not serious	Serious	Not serious	Serious	Not suspected	⨁⨁◯◯ Low
Enucleation time	5	Not serious	Serious	Not serious	Not serious	Not suspected	⨁⨁⨁◯ Moderate
Morcellation time	5	Not serious	Serious	Not serious	Not serious	Not suspected	⨁⨁⨁◯ Moderate

High: ⨁⨁⨁⨁; Moderate: ⨁⨁⨁◯; Low: ⨁⨁◯◯; Very low: ⨁◯◯◯.

## Data Availability

All data generated or analyzed during this study are included in this article. Further enquiries can be directed to the corresponding author.

## Supplementary Materials

The following supporting information can be downloaded at https://www.mdpi.com/article/10.3390/jcm15176661/s1, Table S1: PRISMA 2020 Checklist. Table S2: Detailed Electronic Search Strategies.
