# Supplementary material for: Comparative Effectiveness and Safety of Thulium Laser Enucleation and Holmium Laser Enucleation for BPH: Systematic Review and Meta-Analysis of Randomized Controlled Trials
Source: J Clin Med. 2026 Aug 28;15(17):6661. doi: 10.3390/jcm15176661 (PMC13565839; doi:10.3390/jcm15176661)
Supplement: Supplementary file 1 [file jcm-15-06661-s001.zip › Table S2 jcm-4446954-supplementary.pdf]

Comparative Effectiveness and Safety of Thulium Laser Enucleation and Holmium Laser Enucleation for BPH: Systematic Review and Meta-Analysis of Randomized Controlled Trials

Supplementary Table S2. Detailed Electronic Search Strategies

Database / platform	Search strategy
**PubMed**	**Search date:**<br>**Records retrieved:**<br>(<br>"Prostatic Hyperplasia"[Mesh]<br>OR "benign prostatic hyperplasia"<br>OR "benign prostatic hypertrophy"<br>OR "benign prostatic enlargement"<br>OR "prostatic hyperplasia"<br>OR "prostate hyperplasia"<br>OR "prostatic hypertrophy"<br>OR BPH<br>OR "benign prostatic obstruction"<br>OR "lower urinary tract symptom"<br>OR "lower urinary tract symptoms"<br>OR LUTS<br>)<br>AND<br>(<br>"Laser Therapy"[Mesh]<br>OR "Lasers, Solid-State"[Mesh]<br>OR "holmium laser enucleation of the prostate"<br>OR "holmium laser enucleation"<br>OR "holmium enucleation"<br>OR HoLEP<br>OR "thulium laser enucleation of the prostate"<br>OR "thulium laser enucleation"<br>OR ThuLEP<br>OR "thulium fiber laser enucleation of the prostate"<br>OR "thulium fibre laser enucleation of the prostate"<br>OR "thulium fiber laser enucleation"<br>OR "thulium fibre laser enucleation"<br>OR ThuFLEP<br>OR "thulium laser vapoenucleation"<br>OR ThuVEP<br>OR (<br>(holmium[tiab] OR thulium[tiab] OR "thulium fiber"<br>OR "thulium fibre"[tiab] OR "Tm:YAG")<br>AND<br>(enucleat*[tiab] OR vapoenucleat*])<br>)<br>)<br>AND<br>(<br>"Randomized Controlled Trial"[Publication Type]<br>OR "Controlled Clinical Trial"[Publication Type]<br>OR random*<br>OR RCT<br>OR "controlled trial"

Database / platform	Search strategy
	OR "clinical trial"<br>)<br>NOT (animals[mh] NOT humans)
**Embase**	**Search date:**<br>**Records retrieved:**<br>'prostate hypertrophy'<br>OR 'benign prostatic hyperplasia'<br>OR 'benign prostatic hypertrophy'<br>OR 'benign prostatic enlargement'<br>OR 'prostatic hyperplasia'<br>OR bph<br>OR 'benign prostatic obstruction'<br>OR 'lower urinary tract symptom'<br>OR 'lower urinary tract symptoms'<br>OR luts<br>)<br>AND<br>('laser therapy'<br>OR 'solid state laser'<br>OR 'holmium laser enucleation of the prostate'<br>OR 'holmium laser enucleation'<br>OR holep<br>OR 'thulium laser enucleation of the prostate'<br>OR 'thulium laser enucleation'<br>OR thulep<br>OR 'thulium fiber laser enucleation of the prostate'<br>OR 'thulium fibre laser enucleation of the prostate'<br>OR 'thulium fiber laser enucleation'<br>OR 'thulium fibre laser enucleation'<br>OR thuflep<br>OR 'thulium laser vapoenucleation'<br>OR thuvep<br>OR ((holmium OR thulium OR 'thulium fiber' OR 'thulium fibre'<br>OR 'Tm:YAG')<br>(enucleat* OR vapoenucleat*)))<br>AND<br>('randomized controlled trial'<br>OR 'controlled clinical trial'<br>OR random*<br>OR rct:<br>OR 'controlled trial'<br>OR 'clinical trial'<br>)NOT ('animal' NOT 'human')
**Cochrane Library**	**Search date:**<br>**Records retrieved:**<br>#1 MeSH descriptor: [Prostatic Hyperplasia]<br><br>#2 ("benign prostatic hyperplasia" OR "benign prostatic hypertrophy"<br>OR "benign prostatic enlargement" OR "prostatic hyperplasia"<br>OR "prostate hyperplasia" OR BPH OR "benign prostatic obstruction"<br>OR "lower urinary tract symptom" OR "lower urinary tract symptoms"<br>OR LUTS):<br><br>#3 MeSH descriptor: [Laser Therapy]

Database / platform	Search strategy
	#4 MeSH descriptor: [Lasers, Solid-State]<br><br>#5 ("holmium laser enucleation of the prostate"<br>OR "holmium laser enucleation" OR "holmium enucleation" OR HoLEP<br>OR "thulium laser enucleation of the prostate"<br>OR "thulium laser enucleation" OR ThuLEP<br>OR "thulium fiber laser enucleation of the prostate"<br>OR "thulium fibre laser enucleation of the prostate"<br>OR "thulium fiber laser enucleation"<br>OR "thulium fibre laser enucleation" OR ThuFLEP<br>OR "thulium laser vapoenucleation" OR ThuVEP<br>OR ((holmium OR thulium OR "thulium fiber" OR "thulium fibre"<br>OR "Tm:YAG") NEAR/3 (enucleat* OR vapoenucleat*)))<br><br>#6 #1 OR #2<br>#7 #3 OR #4 OR #5<br>#8 #6 AND #7
**Web of Science**	**Search date:**<br>**Records retrieved:**<br>TS=("benign prostatic hyperplasia" OR "benign prostatic hypertrophy"<br>OR "benign prostatic enlargement" OR "prostatic hyperplasia"<br>OR "prostate hyperplasia" OR BPH OR "benign prostatic obstruction"<br>OR "lower urinary tract symptom*" OR LUTS)<br>AND<br>TS=("holmium laser enucleation of the prostate"<br>OR "holmium laser enucleation" OR "holmium enucleation" OR HoLEP<br>OR "thulium laser enucleation of the prostate"<br>OR "thulium laser enucleation" OR ThuLEP<br>OR "thulium fiber laser enucleation of the prostate"<br>OR "thulium fibre laser enucleation of the prostate"<br>OR "thulium fiber laser enucleation"<br>OR "thulium fibre laser enucleation" OR ThuFLEP<br>OR "thulium laser vapoenucleation" OR ThuVEP<br>OR ((holmium OR thulium OR "thulium fiber" OR "thulium fibre"<br>OR "Tm:YAG") NEAR/3 (enucleat* OR vapoenucleat*)))<br>AND<br>TS=(random* OR RCT OR "controlled trial" OR "clinical trial")
**Scopus**<br>*Elsevier*	**Search date:**<br>**Records retrieved:**<br>TITLE-ABS-KEY("benign prostatic hyperplasia" OR "benign prostatic hypertrophy"<br>OR "benign prostatic enlargement" OR "prostatic hyperplasia"<br>OR "prostate hyperplasia" OR BPH OR "benign prostatic obstruction"<br>OR "lower urinary tract symptom*" OR LUTS)<br>AND<br>TITLE-ABS-KEY("holmium laser enucleation of the prostate"<br>OR "holmium laser enucleation" OR "holmium enucleation" OR HoLEP<br>OR "thulium laser enucleation of the prostate"<br>OR "thulium laser enucleation" OR ThuLEP<br>OR "thulium fiber laser enucleation of the prostate"<br>OR "thulium fibre laser enucleation of the prostate"<br>OR "thulium fiber laser enucleation"<br>OR "thulium fibre laser enucleation" OR ThuFLEP<br>OR "thulium laser vapoenucleation" OR ThuVEP

Database / platform	Search strategy
	OR ((holmium OR thulium OR "thulium fiber" OR "thulium fibre" OR "Tm:YAG") W/3 (enucleat* OR vapoenucleat*)))<br>AND<br>TITLE-ABS-KEY(random* OR RCT OR "controlled trial" OR "clinical trial")
**CBM / SinoMed**	**Search date:**<br>**Records retrieved:**<br>#1 prostatic hyperplasia OR benign prostatic hyperplasia OR benign prostatic hypertrophy OR prostatic hypertrophy OR benign prostatic obstruction OR lower urinary tract symptoms OR BPH OR LUTS<br><br>#2 holmium laser enucleation of the prostate OR transurethral holmium laser enucleation of the prostate OR holmium laser enucleation OR HoLEP OR thulium laser enucleation of the prostate OR transurethral thulium laser enucleation of the prostate OR thulium laser enucleation OR ThuLEP OR thulium fiber laser enucleation of the prostate OR thulium fibre laser enucleation of the prostate OR ThuFLEP OR thulium laser vapoenucleation OR ThuVEP<br><br>#3 randomized controlled trial OR randomized controlled OR randomized OR random allocation OR controlled trial OR RCT<br><br>#4 #1 AND #2 AND #3

Abbreviations: BPH, benign prostatic hyperplasia; CBM, Chinese Biomedical Literature Database; CENTRAL, Cochrane Central Register of Controlled Trials; HoLEP, holmium laser enucleation of the prostate; LUTS, lower urinary tract symptoms; RCT, randomized controlled trial; ThuFLEP, thulium fiber laser enucleation of the prostate; ThuLEP, thulium laser enucleation of the prostate; ThuVEP, thulium laser vapoenucleation of the prostate. For CBM/SinoMed, English translations of the original Chinese search terms are presented.
